# First-generation medical school applicants: A quantitative study designed to identify areas of educational support

**DOI:** 10.1371/journal.pone.0342616

**Published:** 2026-07-17

**Authors:** Bethsabe Romero, Amanda K. Burbage

**Affiliations:** 1 Eastern Virginia Medical School, Macon & Joan Brock Virginia Health Sciences at Old Dominion University, Norfolk, Virginia, United States of America; 2 Medical & Health Professions Education (PhD, MHPE, Certificate), Macon & Joan Brock Virginia Health Sciences at Old Dominion University, Norfolk, Virginia, United States of America; UCSF: University of California San Francisco, UNITED STATES OF AMERICA

## Abstract

First-generation (First Gen) students are unique medical school applicants. Due to their lived experience, they approach patient care by prioritizing trust, comfort and understanding. They have proven ability to overcome obstacles and were found to be more resilient than their continuing generation (Cont Gen) peers. Despite these notable attributes, they face unique challenges in gaining medical school acceptance. There are very few quantitative studies examining this student subpopulation, and our study identifies characteristics of first-generation medical school applicants while highlighting areas of needed support. This cross-sectional study used deidentified Application and Matriculating Student Questionnaire survey data that was obtained from the AAMC for 286,656 total applicants for the 2017–2024 period, in which 12.5% identified as First Gen. Descriptive statistics, Mann-Whitney U, independent-samples t*-*tests, and Chi-square tests were performed to define medical school applicant characteristics, evaluate applicants’ motivations to study medicine, and obstacles to medical school acceptance. All First Gen applicants who reported socioeconomic status (SES) come from low SES backgrounds and are motivated to study medicine by establishing a secure future, eliminating career debt, and affecting social change. First Gen applicants had lower acceptance (43% vs. 57%, p < 0.001) and matriculation rates (42% vs. 56%, p < 0.001). Obstacles to admission include First Gen and low SES statuses, age at matriculation, and time spent in family obligations, each significantly associated with lower acceptance rates (p < 0.001). First Gen medical school applicants and matriculants face disproportionate challenges as they navigate careers in medicine. This diverse group of medical school applicants has much to contribute to the future of medicine. Our study highlights a continued need for academic intervention to reduce barriers in the pre-matriculation stage and throughout medical training for First Gen medical school applicants and matriculants.

## Introduction

First-generation (First Gen) students are a diverse subpopulation of medical school applicants who face unique challenges in gaining medical school acceptance. The Association of American Medical Colleges (AAMC) defines First Gen students as those whose parents have not earned an associate’s degree or higher [1]. First Gen students have increased levels of stress, fatigue, financial worries, lower quality of life, and decreased social support [1–8]. They may struggle academically because they lack cultural capital, and experience financial insecurity and ethnic discrimination [2]. Despite these challenges First Gen students have proven resilience, demonstrating greater ability to draw on internal resources and persist through academic and personal challenges, than their continuing generation (Cont Gen) peers who have at least one parent with a post-secondary degree [1,2,9,10].

Understanding the role of First Gen applicants in medical education is particularly important given broader workforce challenges in medicine. The AAMC estimates a physician shortage of between 54,100–139,000 physicians by 2033 [11–12]. This shortage will disproportionately affect rural and low-income communities [11], demonstrating a great need to train physicians who can address healthcare disparities and have a vested interest in improving health inequity. First-generation (First Gen) students have insight into healthcare disparities due to their lived experience [3,13]. Many have family members who are unfamiliar with the medical field leading to anxiety, fear, and distrust of healthcare providers [3]. This frames their approach to patient care by prioritizing trust, comfort, and understanding [2–3]. First Gen medical students admit to setting aside personal interests “for the greater good” resulting in empathetic and culturally competent care [3,13]. These non-cognitive factors shape empathetic and compassionate physicians but are not consistently addressed in admissions processes nor developed in medical school curricula [14].

Several studies have called for an improvement in the medical education environment and resources for First Gen students to mitigate academic challenges [1,5,6,11,13]. A few studies have evaluated First Gen medical students’ level of support and sense of belonging, [3–4] while some universities have established support programs for First Gen students [3,11]. Although First Gen students represent a substantial proportion of students enrolled at U.S. four-year colleges and universities, they remain underrepresented in medical education. Yet a large-scale quantitative study understanding First Gen medical school applicants is lacking [4,8,15]. To address this gap, we examined the demographics of First Gen and Cont Gen applicants and assessed the importance of motivating factors to pursue a career in medicine. We further examined the association of challenges with medical school acceptance for First Gen medical school applicants. This study addressed the following research questions: (1) What is the impact of career motivators such as career debt, career high income, career social status, and career secure future as motivation to study medicine? (2) What is the impact of obstacles such as First Gen status, socioeconomic status (SES), age at matriculation, and family obligation on medical school acceptance?

## Methods

### Study design & sample

This retrospective cross-sectional analysis of applicant-level data from the 2017–2024 AAMC application cycles examined First Gen and Cont Gen medical school applicants and their career motivations. De-identified Application and Matriculating Student Questionnaire survey data was obtained in August 2025 from the AAMC for 286,656 total applicants for the 2017–2024 period. The original dataset included application year records with potential for multiple cycles. The present analysis was conducted after data cleaning and deduplication at the individual level, retaining the final application record. Within the cohort, 35,962 applicants identified as First Gen. Analysis was performed using IBM SPSS Statistics for Windows, version 29.0 (IBM Corp., Armonk, N.Y., USA).

### Variables

Demographic data were used to understand the First Gen population. Socioeconomic status (SES) categories were defined by the AAMC using parental occupation–based classifications (E01–E05) and were derived from applicant-reported data. Rurality was determined by the AAMC based on applicants’ legal residency and classified as residence outside a Standard Metropolitan Statistical Area (SMSA) with fewer than 50,000 residents. Race/ethnicity categories were derived from AAMC Identity Array codes and analyzed as mutually exclusive categories. All demographic and survey variables were self-reported by applicants through the AAMC application system. Academic variables included binary responses (yes or no) about acceptance and matriculation into medical school. Career variables such as career social change, career debt, career high income, career social status, and career secure future were hypothesized a priori as motivators to study medicine. Applicants’ responses were collected using the following survey question: “When thinking about your career path after medical school, how important are the following considerations?”. Answer choices included “not important”, “somewhat important”, “very important”, and “essential”. Answers were transformed into numeric values for ordinal analysis: 1 = not important, 2 = somewhat important, 3 = very important, 4 = essential. First-generation status (yes or no), SES status (high or low), spending time between application and matriculation fulfilling family obligations (yes or no), and age at matriculation (<22 years of age, 22–27, > 27 years of age) were evaluated as proxies for obstacles for medical school acceptance. Age at matriculation was used as a surrogate for age.

### Data analysis

Frequencies and percentages were used to summarize categorical data, while measures of central tendency and distribution were used to characterize continuous demographic data. Mann Whitney U tests were performed to assess the importance of career variables as motivators to study medicine. Chi-square tests were performed to evaluate the association between First Gen status and Cont Gen status of applicants with medical school acceptance and matriculation. Chi-square tests were also used to assess the association of obstacles such as low SES, family obligation and age at matriculation with medical school acceptance in First Gen and Cont Gen applicants. Independent-samples t-tests were used to compare GPAs and MCAT scores. Due to the large sample size, parametric analyses were considered robust to modest deviations from normality, while nonparametric Mann Whitney U tests were used for analysis of ordinal motivation variables. Assumptions for associated tests were evaluated for statistical appropriateness of the test before proceeding. Statistical significance level for all analyses was determined at *p*-values <0.05.

### Ethical approval

Ethical approval was waived for this project which does not involve human subjects by Eastern Virginia Medical School IRB Committee (IRB# 23–01-NH- 0020) on January 9, 2023.

## Results

Descriptive statistics of demographic data comparing First Gen and Cont Gen applicants are listed in Table 1. In summary, a greater proportion of First Gen and Cont Gen applicants were female. The white student ethnic group contained the greatest number of applicants in both First Gen and Cont Gen cohorts. Most applicants (First Gen and Cont Gen) were permanent residents, from non-rural areas, and between the ages of 22–27. First Gen applicants were primarily from low SES backgrounds while Cont Gen applicants were from high SES backgrounds *X*^*2*^ (6, N = 286,656) = 1.10 x 10^5^, Cramer’s V = 0.44, *p* ≤ 0.001. Approximately 60% of First Gen applicants identified as underrepresented minorities (URM) while 49% of Cont Gen applicants identified as URM.

**Table 1:** Demographics for medical school applicants from 2017-2024 (N = 286,656), (SES = socioeconomic status, URM = underrepresented minority).

		First Generation			Continuing Generation		
		*n*	*% within First Gen*	*% total*	*n*	*% within Cont Gen*	*% total*
**Sex**	*Female*	20400	56.9	7.1	127672	55.9	44.5
	*Male*	15481	43.1	5.4	100831	44.1	35.2
**Race**	*White*	11194	31.2	3.9	111372	48.6	38.9
	*Asian*	8191	22.8	2.9	71941	31.4	25.1
	*Hispanic*	7188	20.0	2.5	18697	8.2	6.5
	*Black*	5694	15.9	2.0	20407	8.9	7.1
	*American Indian*	332	0.9	0.1	1481	0.6	0.5
	*Pacific Islander*	79	0.2	0.0	304	0.1	0.1
	*Unknown*	1216	3.4	0.4	4854	2.1	1.7
**Geography**	*Rural*	2261	6.5	0.8	476	0.2	0.2
	*Not Rural*	32590	93.5	11.4	209111	99.8	73.0
**SES**	*Low (E01-E02)*	28118	100.0	9.8	36706	17.4	12.8
	*High (E03-E05)*	0	0.0	0.0	174819	82.6	61.0
**URM**		21484	59.7	7.5	112830	49.3	39.4
**Permanent Resident**	*yes*	34662	96.3	12.1	222805	97.3	77.7
	*no*	1340	3.7	0.5	6251	2.7	2.2
**Age at Matriculation**	*<22*	834	2.3	0.3	10635	4.6	3.7
	*22-27*	27695	77.0	9.7	197665	86.3	69.0
	*>27*	7433	20.7	2.6	20756	9.1	7.2

Note: Female, Male, and No Response/Decline to Answer were the available response options for Sex/Gender. Race categories were derived from AAMC Identity Array codes and collapsed into mutually exclusive umbrella categories for analysis. Rurality was determined from applicant legal residency and defined as residence outside a Standard Metropolitan Statistical Area (SMSA) with fewer than 50,000 residents. Low SES included service and unskilled households with bachelor’s or less than bachelor’s educational attainment (AAMC E01–E02), while High SES included executive/professional households with bachelor’s or graduate educational attainment (AAMC E03–E05). Percentages were calculated using available-case denominators. Missing responses and categories representing less than 1% of the data were excluded from analysis. Continuous variables contained less than 25% missing data.

Application and matriculation data are listed in Table 2. Chi-square tests of independence showed that the association between First Gen status and medical school acceptance is significant *X*^*2*^ (2, N = 286,656) = 3,026, Cramer’s V = 0.10, *p* ≤ 0.001, and the association between First Gen status and matriculation is also significant *X*^*2*^ (2, N = 286,656) = 2,933, Cramer’s V = 0.10, *p* ≤ 0.001. First Gen students were less likely to gain medical school acceptance (First Gen = 43%, Cont Gen = 57%) and, among those accepted, less likely to matriculate (First Gen = 42%, Cont Gen = 56%) compared to Cont Gen students. First Gen medical school applicants submitted fewer total applications, received fewer total acceptances, and had lower GPAs and MCAT scores compared to Cont Gen students. Independent-samples t-tests showed a significant difference in GPAs between First Gen (*M* = 3.51, *SD* = 0.4) and Cont Gen (*M* = 3.64, *SD* = 0.3) medical school applicants *t*(174722)=57.6, *p* ≤ 0.001, *d* = 0.3. Statistically significant differences were also seen for MCAT scores between First Gen (*M* = 501.6, *SD* = 10.0) and Cont Gen (*M* = 507.4, *SD* = 9.3) applicants *t*(174722)=86.1, *p* ≤ 0.001, *d* = 0.4.

**Table 2:** Application and matriculation data for First Gen and Cont Gen applicants from 2017-2024.

		First Generation			Continuing Generation		
		*n*	*% within First Gen*	*% total*	*n*	*% within Cont Gen*	*% total*
Accepted	Yes	15597	43.3	5.9	130371	56.9	49.2
	No	20365	56.7	7.7	98685	43.1	37.2
Matriculated Among Accepted Applicants	Yes	15182	42.2	5.7	127452	55.6	48.1
	No	20780	57.8	7.9	101604	44.4	38.3
Accepted	Female	8099	52.1	5.6	71378	54.9	49.0
	Male	7456	47.9	5.1	58705	45.1	40.3
Matriculated Among Accepted Applicants	Female	7861	51.9	5.5	69680	54.8	48.9
	Male	7282	48.1	5.1	57504	45.2	40.5
Average # Applications		17.0 ± 13.0	Median(IQR): 16 (17)		18.0 ± 13.7	Median(IQR): 17 (19)	
Average # Acceptances		0.9 ± 1.7	Median(IQR): 0 (1)		1.1 ± 1.6	Median(IQR): 1 (1)	
Average GPA		3.51 ± 0.4			3.64 ± 0.3		
Average MCAT Score		501.6 ± 10.0			507.4 ± 9.3		

Note: Acceptance analyses included the full applicant sample. Matriculation analyses were limited to applicants who received at least one medical school acceptance. The table excludes missing values (no missing categorical data, < 25% missing data for continuous variables), and categories representing less than 1% of the data were excluded from analysis. Continuous variables are presented as mean ± standard deviation and median (interquartile range) when distributions were non-normal.

Chi-square test results for the association of obstacles with medical school acceptance are listed in Table 3. The associations between acceptance and obstacles (First Gen status, SES status, age at matriculation, and family obligation) were significant for First Gen and Cont Gen applicants. Among all applicants who gained acceptance and reported SES, 61% indicated they were from a high SES background and 48% were from a low SES group. All First Gen applicants who reported SES are from low SES backgrounds, while 76% of Cont Gen applicants are from high SES backgrounds. FG applicants were more likely to be older than 27 years at matriculation compared to CG applicants (21% vs. 9.1%), and applicants older than 27 years had lower acceptance rates overall. Family obligation was also associated with lower acceptance rates in both cohorts.

**Table 3:** Obstacles for medical school acceptance.

First Gen Status and Acceptance					
		% Accepted	% Not Accepted	Asym. Sig.	df
First Gen		43	57		
Continuing Gen		57	43		
Pearson Chi-Square		3026		<0.001	1
Cramer’s V		0.103		<0.001	
**SES and Acceptance**					
Low SES (E01-E02)		48	52		
High SES (E03-E05)		61	39		
Pearson Chi-Square		10339		<0.001	3
Cramer’s V		0.179		<0.001	
**SES and Matriculation**					
Pearson Chi-Square		10644		<0.001	3
Cramer’s V		0.182		<0.001	
**Age at Matriculation and Acceptance**					
*First Gen*	Younger than 22	2	2		
	22-27	82	74		
	Older than 27	16	24		
	Pearson Chi-Square	725		<0.001	7
	Cramer’s V	0.142		<0.001	
*Continuing Gen*	Younger than 22	6	3		
	22-27	88	84		
	Older than 27	6	13		
	Pearson Chi-Square	7714		<0.001	7
	Cramer’s V	0.184		<0.001	
**Family Obligation and Acceptance**					
*First Gen*	Family Obligation	21	<1		
	No Obligation	31	<1		
	Pearson Chi-Square	13793		<0.001	2
	Cramer’s V	0.619		<0.001	
*Continuing Gen*	Family Obligation	9	<1		
	No Obligation	37	<1		
	Pearson Chi-Square	61081		<0.001	2
	Cramer’s V	0.516		<0.001	

Note: Analyses were conducted using the final deduplicated analytic sample (N = 286,656).The table excludes missing values (no missing categorical data, < 25% missing data for continuous variables), and categories representing less than 1% of the data were excluded from analysis. Asym. Sig. = 2-sided Asymptotic Significance, df = degrees of freedom.

Independent samples Mann-Whitney U test results and descriptive statistics for First Gen and Cont Gen applicants’ motivations to study medicine are listed in Tables 4 and 5. Trends were similar between First Gen applicants and Cont Gen applicants for all career motivators; however, the importance of career motivators differed between applicant groups. First Gen applicants ranked the importance of “social change” and minimizing “career debt” higher than Cont Gen applicants. The greatest difference between First Gen and Cont Gen applicants was selecting medicine as a career to minimize “career debt”. There was no significant difference between First Gen and Cont Gen applicants in selecting medicine as a career to improve social status.

**Table 4:** First gen and Cont gen motivations for studying medicine.

	N	Median (IQR)		Mean		Mann-Whitney U	Z	*p*
		*First Gen*	*Cont Gen*	*First Gen*	*Cont Gen*			
**Social Change**	94277	3 (3-4)	3 (2-4)	3.11	2.92	3.7 x 10^8^	−21.3	<0.001
**High Income**	94250	2 (2-3)	2 (2-3)	2.42	2.35	4.0 x 10^8^	−7.4	<0.001
**Social Status**	94172	2 (1-2)	2 (1-2)	1.72	1.72	4.2 x 10^8^	−0.2	0.808
**Secure Future**	94289	4 (3-4)	3 (3-4)	3.43	3.37	4.0 x 10^8^	−8.6	<0.001
**Debt**	94145	4 (3-4)	3 (3-4)	3.35	3.10	3.6 x 10^8^	−24.2	<0.001

Note: Median and mean values are representative of importance of variables: 1 = not important, 2 = somewhat important, 3 = very important, 4 = essential. The table excludes missing values (no missing categorical data, < 25% missing data for continuous variables), and categories representing less than 1% of the data were excluded from analysis.

**Table 5:** Motivations for career path after medical school.

		N	%Not Important	%Somewhat Important	%Very Important	% Essential
**First Gen**	Social Change	9928	2.8	20.4	39.8	37.0
	Career Debt	9913	4.8	10.5	29.2	55.5
	High Income	9916	10.4	46.8	33.1	9.7
	Social Status	9918	44.2	42.0	11.3	2.6
	Stable Future	9926	0.6	8.5	38.2	52.6
**Continuing** **Gen**	Social Change	84349	4.2	26.5	41.9	27.3
	Career Debt	84232	9.8	14.7	31.2	44.3
	High Income	84334	11.3	49.2	32.2	7.3
	Social Status	84254	43.8	42.5	11.7	2.0
	Stable Future	84363	0.7	9.7	41.7	48.0

Note: The table excludes missing values (no missing categorical data, < 25% missing data for continuous variables), and categories representing less than 1% of the data were excluded from analysis.

## Discussion

### First-generation medical school applicants continue to be underrepresented

The term “first-generation” is considered a key DEI marker and is sometimes used interchangeably with the term URM [2]. However, not all first-generation medical school applicants are URM and 31% identify as “white”. Most First Gen applicants were found to be demographically similar to Cont Gen applicants but in decreased numbers. There were considerable differences in the number of Hispanic and black applicants between Cont Gen and First Gen cohorts. The number of Hispanic and black applicants were approximately 6-times and 5-times fewer than the white ethnic group in the Cont Gen cohort, and 1.6-times and 2-times fewer compared to the white ethnic group in the First Gen cohort. These findings demonstrate persistent underrepresentation of Hispanic and Black applicants within the present applicant pool despite ongoing national efforts to improve representation in medicine.

Previous studies found that 20% of medical students identify as First Gen and half of these students are URM [2–4,16]. The AAMC estimates that 12.4% of 2021–2022 matriculants to MD-granting medical schools are First Gen college students, an increase from 10.8% in 2018–2019. Taking a longitudinal view, our study found that 10.6% of matriculants were First Gen students (total matriculants = 142,634), while 12.5% of total applicants identified as First Gen (total applicants = 286,656). Among students who identified as First Gen, approximately 60% of applicants also identified as URM which is greater than previously reported. Our findings differ from previous studies and demonstrate that First Gen students continue to be underrepresented in medical schools.

### Medical school acceptance and matriculation obstacles persist

First Gen medical school applicants and matriculants face disproportionate challenges as they navigate careers in medicine [1–10]. We evaluated the association between medical school acceptance and obstacles such as First Gen status, SES status, age at matriculation and family obligation. As shown in Tables 2 and 3, each of these variables was significantly associated with lower acceptance rates. Even when examined independently, First Gen status was associated with decreased acceptance and matriculation relative to continuing-generation applicants.

Lower acceptance and matriculation may be due to several factors. First Gen medical school applicants submitted fewer total applications (17 vs.18) and had lower GPAs (3.51 vs. 3.64) and MCAT scores (502 vs. 507) compared to Cont Gen students. First Gen applicants face greater discouragement from pre-medical advisors [8] and are more likely to struggle with financial insecurity [1,3,6]. First Gen applicants from low-income communities have fewer resources, and decreased access to high-quality school systems leading to fewer educational opportunities [11].

One of the greatest challenges First Gen applicants experience is financial insecurity [3,6]. Previous studies have asserted that First Gen applicants come from families with lower total parental income [1,3,11]. Our study confirms this and provides additional data: all (100%) First Gen applicants who reported SES came from low SES backgrounds. Strikingly, none of the First Gen applicants reported high SES backgrounds. In contrast, 83% of Cont Gen students came from high SES backgrounds while 17% of Cont Gen applicants came from low SES backgrounds. This is concerning because there are statistically significant associations between low SES status and medical school acceptance and matriculation. Twenty-two percent of matriculants came from low SES backgrounds while 70% of matriculants came from high SES backgrounds. Over the past 45 years, 73%−79% of medical school matriculants have come from top income quintiles [16–18], and there is a positive association between median family income and higher rates of medical school acceptance and matriculation [11]. Further, the lack of social mobility in medicine makes it an unreachable profession for many from underprivileged backgrounds [19].

In addition to lacking financial resources, First Gen applicants typically lack cultural capital. Cultural capital is defined as “knowledge, skills, education, or other social advantages that permit educational success” [2,3,20]. Cultural capital can be acquired with increased financial resources and is less accessible to First Gen medical school applicants from low-income communities. First Gen medical students’ lack of cultural capital leads to less knowledge about study resources, research opportunities, residency applications, and residency program pathways [2,19]. In competitive specialties, it is becoming increasingly important to connect with faculty members at programs of interest. Many of these interactions include informal communication obtained through mentors or home program faculty members. First Gen medical students that lack cultural capital are at a significant disadvantage, especially when faced with residency application challenges. While our study did not directly evaluate cultural capital, cultural capital is closely tied to greater financial resources [20], and this may provide another barrier to success for First Gen applicants and medical students.

We evaluated increasing age at matriculation as a potential obstacle to medical school admission since previous studies indicated that First Gen medical students are more likely to be older and have family obligations [1,3,20]. While a larger percentage of First Gen applicants are older than 27 years of age compared to Cont Gen applicants (21% vs. 9%), most First Gen and Cont Gen matriculants are between the ages of 22–27 (77% of First Gen and 86% of Cont Gen). Increased age at application (>27 years of age) is associated with decreased acceptance. Among applicants who are older than 27 years of age, 67% of First Gen and 61% Cont Gen applicants did not gain acceptance. Interestingly, a previous study found that while younger students had higher undergraduate GPAs compared to older students (>28 years of age), there was no difference in medical licensing exam performance [21].

Previous studies indicated that First Gen applicants struggled to balance academics with family obligations [1,3,20]. We discovered that 9% of First Gen applicants and 5% of Cont Gen applicants had family obligations. When we evaluated medical acceptance rates, we found that 21% of First Gen applicants and 9% of Cont Gen applicants with family obligations gained acceptance. Among applicants without family obligations, 31% of First Gen and 37% of Cont Gen applicants gained acceptance. Time spent on family obligations leaves less time for academic preparation and extracurricular activities. Engaging in medical research and clinical volunteering demonstrates a deeper commitment to studying medicine and provides evidence of competencies necessary for medical school success [22]. We found that time allocated to family obligations is a barrier to acceptance for all applicants.

Despite numerous barriers, First Gen applicants are driven to succeed in medicine. We evaluated the importance of motivating factors such as social change, high income, social status, secure future, and paying career debt. Overall trends were similar for both groups. However, the percentage of First Gen applicants that ranked social change as an “essential” motivating factor to study medicine was greater than the percentage of Cont Gen applicants. Thirty-seven percent of First Gen applicants considered social change “essential” compared to 27% of Cont Gen applicants. Our results align with the literature indicating that First Gen students are more likely to want to care for underserved populations and work in underserved areas [3,13]. Considering the anticipated physician shortage in underserved communities, support of First Gen students in gaining admission and throughout medical school should be prioritized.

First Gen applicants are also motivated to study medicine because of the potential to have a financially stable career. Paying career debt was considered “essential” by 55% First Gen applicants and 44% of Cont Gen applicants. Obtaining a stable future by studying medicine was considered “essential” by 53% of First Gen applicants and 48% of Cont Gen applicants. When we evaluate all motivating variables, we found that influencing social change, obtaining a secure future, and paying career debt had the highest means among First Gen applicants.

### Need for targeted areas of support

Our study found key differences in First Gen medical school applicant data compared to previous literature. First Gen applicants contribute to medical student and physician diversity which is correlated to improved educational experiences and improved health care outcomes [2,23–26]. Moreover, medical students who train with diverse peers are more prepared to meet the needs of diverse patients [27]. First Gen medical school students’ experiences may be more similar to their patients’ life experiences, leading to improved patient-provider relationships. Because First Gen students can be valuable contributors to diversity as health care providers and physician peers, it is imperative to support them through medical school and residency training. However, to ensure their success in medicine, we need to reduce application and admission barriers and better understand this unique student population and the challenges they face.

Several medical school programs have established formal support programs for First Gen medical students, and holistic medical school admissions processes are now widely accepted [4,5,21,27–30]. These changes have allowed First Gen applicants to gain acceptance, but do not ensure that First Gen students will be successful once they gain admission [10,13]. Discussions between members of the Committee on Student Diversity Affairs (COSDA) of the AAMC led to the development of the Integrated Holistic Student Affairs (IHSA) Model in 2020 [30]. This model provides a framework to better support First Gen medical students and URM by shifting from a deficit-centered approach to a student-centered approach that is empowering and equitable [30]. A handful of medical schools have adopted this model, and further studies are needed to assess its success with First Gen medical students. Further studies are also needed to determine why the model hasn’t been adopted by all medical schools.

Insufficient institutional support and further studies on First Gen applicants are potential areas of intervention. Havemann *et al.* have advocated for free tutoring, mentorship, and academic support specialists for all students [10]. First Gen medical students lacking educational support and cultural capital would benefit from medical school systems that make implicit expectations more transparent. The AAMC provides online “Tools and Resources for First-Generation Medical School Students” which provides resources for academic, professional, emotional and financial support [31]. Although the present study focused on U.S. medical school applicants, Harrison *et al.* demonstrate similar interests are being explored in the United Kingdom, having established a large cohort study of medical school applicants who are first in family that will identify applicant experiences, the effect of sociodemographics, and the overall effect on the workforce [19]. Admirable efforts have been made to support and study First Gen applicants; however, the data indicate First Gen medical school applicants need continued academic support, continued study and a reduction in barriers to admission and matriculation.

## Strengths and limitations

One limitation of our study is that cross sectional studies are insufficient for causal claims, however, using generational status as a comparative control helps us better understand specific sub populations of applicants and matriculants. We evaluated some obstacles for First Gen applicants but there are several that were not addressed which have been supported by other studies. Data are self-reported and subject to issues of interpretation or measurement error. Finally, several other pre-application and pre-matriculation factors are excluded from this analysis including baccalaureate preparatory experiences, undergraduate advising structures, and invitations to submit secondary applications or interview with a prospective school. Additional structural barriers may also contribute to disparities in medical school access, including reduced availability of Advanced Placement coursework, lower participation in premedical enrichment opportunities, and unequal access to institutional advising resources. Although most of these data were not available, future researchers may consider a smaller but more comprehensive analysis which accounts for these factors.

## Conclusions

First Gen medical school applicants and matriculants face disproportionate challenges as they navigate careers in medicine. Our study represents the largest quantitative study of First Gen applicants and matriculants to date. We have highlighted some of the obstacles that First Gen applicants face such as First Gen status and financial insecurity, and several motivating factors to pursue a career in medicine. First Gen applicants are more resilient than their peers and they significantly increase diversity which is statistically associated with better communication, quality of care, and better health outcomes. Several medical schools have established support programs for First Gen medical students, and our study highlights a continued need for academic intervention to support First Gen medical students.
